# Modulation of CRMP2 via (*S*)-Lacosamide shows therapeutic promise but is ultimately ineffective in a mouse model of CLN6-Batten disease

**DOI:** 10.1042/NS20190001

**Published:** 2019-04-08

**Authors:** Katherine A. White, Jacob T. Cain, Helen Magee, Seul Ki Yeon, Ki Duk Park, Rajesh Khanna, Jill M. Weimer

**Affiliations:** 1 Pediatrics and Rare Diseases Group, Sanford Research, Sioux Falls, SD, U.S.A.; 2 Basic Biomedical Sciences, University of South Dakota Sanford School of Medicine, Vermillion, SD, U.S.A.; 3 Convergence Research Center for Dementia, Brain Science Institute, Korea Institute of Science and Technology, Seoul, South Korea; 4 Department of Pharmacology, University of Arizona, Tucson, AZ, U.S.A.; 5 Department of Pediatrics, University of South Dakota, Sioux Falls, SD, U.S.A.

**Keywords:** Batten disease, CRMP2, Lacosamide, neuronal ceroid lipofuscinosis, repurposed pharmaceuticals

## Abstract

CLN6-Batten disease is a rare neurodegenerative disorder with no cure, characterized by accumulation of lipofuscin in the lysosome, glial activation, and neuronal death. Here we test the therapeutic efficacy of modulating collapsin response mediator protein 2 (CRMP2) activity via *S*-N-benzy-2-acetamido-3-methoxypropionamide ((*S*)-Lacosamide) in a mouse model of CLN6-Batten disease. Promisingly, mouse neuronal cultures as well as *Cln6* patient fibroblasts treated with varying concentrations of (*S*)-Lacosamide showed positive restoration of lysosomal associated deficits. However, while acute *in vivo* treatment enhanced glial activation in 3-month-old *Cln6* mutant mice, chronic treatment over several months did not improve behavioral or long-term survival outcomes. Therefore, modulation of CRMP2 activity via (*S*)-Lacosamide alone is unlikely to be a viable therapeutic target for CLN6-Batten disease.

## Introduction

CLN6-Batten disease is a rare, autosomal recessive, neurodegenerative disorder with no cure. While there are many subforms of Batten disease, the CLN6 variant arises from various mutations in the *CLN6* gene, the most common of which leads to a frameshift mutation and ultimate loss of expression of the CLN6 protein [1]. The function of the transmembrane CLN6 protein is currently unknown, though its localization to the endoplasmic reticulum suggests roles in ER and oxidative stress, autophagy regulation, endocytosis, and neuronal trafficking, among others [2–5]. Interestingly, while CLN6 has not been found to colocalize with any lysosomal markers, loss of CLN6 ultimately leads to classic Batten disease pathology, including accumulation of aggregates in the lysosome (autofluorescent storage material, mitochondrial ATP synthase subunit C (SubunitC)), enhanced glial activation, neuron loss, motor, visual, and memory/learning decline, and ultimately early death. One reason for this may be CLN6’s association with the cytoskeletal collapsin response mediator protein 2 (CRMP2), which plays a role in neuronal polarization, migration, and differentiation [6], and has shown to be reduced in *Cln6* mutant mouse brains [7,8]. Alterations in CRMP2 activity have been implicated in a number of neurological diseases including Alzheimer’s disease, where aggregates of hyperphosphorylated CRMP2 have been found in neurofibrillary tangles, and have been hypothesized to contribute to the death of *Cln6* mutant neurons in culture [7–11].

Here we test the efficacy of *S*-N-benzy-2-acetamido-3-methoxypropionamide ((*S*)-Lacosamide), a CRMP2 modulating functionalized amino acid [12,13], in CLN6-Batten disease. (*S*)-Lacosamide is the presumptive inactive stereoisomer of the clinically used antiepileptic drug (*R*)-Lacosamide (Vimpat®). Treatment with (*R*)-Lacosamide has been shown to be neuroprotective in a number of contexts, specifically reducing apoptosis and glial activation in a rodent model of ischemic stroke, and improving memory and learning in a mouse model of Alzheimer’s disease [14,15]. As (*S*)-Lacosamide has recently been shown to specifically inhibit CRMP2 phosphorylation via cyclin-dependent kinase 5 (Cdk5) [16], we hypothesized that treatment with (*S*)-Lacosamide would prove to be therapeutic in models of CLN6-Batten disease [16]. Ultimately, while there were some positive benefits of (*S*)-Lacosamide on *Cln6* mouse neuronal cultures and *CLN6* patient fibroblasts, *in vivo* treatment with (*S*)-Lacosamide did not have positive functional effects on behavioral or survival outcomes of *Cln6* mutant mice.

## Methods

### Ethics statement/animals

All animal studies were carried out at Sanford Research, Sioux Falls, SD, U.S.A. in strict accordance with National Institutes of Health guidelines and were approved by the Sanford Institutional Animal Care and Use Committee (USDA License 46-R-0009). Wild-type (WT) and homozygous *Cln6-*mutant mice (*Cln6^nclf’^*; JAX stock #003605) on C57BL/6J backgrounds were utilized for all studies and were housed under identical conditions [17]. All animals were genotyped using previously described techniques [18]. Experimenters were blinded to the treatment conditions.

### Cell culture

Human patient fibroblasts were grown from skin punches (*CLN6* samples were young females), and genotype was confirmed with Sanger Sequencing (IRB #03-13-060). *CLN6* mutations included two patient lines with c.486+1G>A, c.184C>T; two patient lines with a homozygotic G>T substitution on exon 7, resulting in M241I mutations; and two patient lines with V148D mutations. Fibroblasts were maintained in DMEM supplemented with 10% fetal bovine serum and 1% penicillin and streptomycin in cell culture treated T75 flasks (Corning). Forty-eight hours prior to imaging and quantitation, cells were trypsinized and re-plated on to 96-well optical bottom plates at a density of 5000 cells per well.

Primary cortical neurons were collected from embryonic day 15.5 WT and *Cln6^nclf^* embryos. In brief, the cortices from WT or *Cln6^nclf^* embryos were subdissected away from the midbrain and hippocampus, taking care to remove the meninges, and were pooled together for enzymatic digestion with papain. Neurons were then suspended in Neural Basal Media supplemented with B27 and glutamine and plated in Laminin coated 96-well plates, with an optical bottom, at a density of 5000 cells per well.

### Drug treatment

Cultures were treated with 2 or 200 μM of (*S*)-Lacosamide for 48 h before analysis. For the acute short-term study, 2-month-old animals (*n*=4/sex) received intraperitoneal (i.p.) injections of either Ringer’s solution or (*S*)-Lacosamide at 20 mg/kg once daily for 7 days. Animals were killed at 3 months of age. For the long-term study, male animals received daily i.p. injections of either Ringer’s solution or (*S*)-Lacosamide at 20 mg/kg from 2 to 5 months of age. Animals were either killed at 11 months of age or kept on for survival analysis. Animals that perished in the study were declared moribund based on weight loss and body condition criteria.

### Live cell imaging and quantitation

Prior to imaging, cells were incubated with either Lysotracker Red (1:10000; Molecular Probes), Mitotracker Red (1:10000; Molecular Probes), or LAMP1 (1:1000; Santa Cruz Biotechnology) and Hoescht (1:10000; Molecular Probes) for 30 min, before being washed two times for 5 min in Hank’s Balanced Salt Solution (HBSS). Cells were then left in HBSS for imaging. Cells were imaged and quantitated using the CellInsight CX7 High Content System (Thermo Fisher) using the Spot Detector Bioapplication (ver. 4). Images were taken at 20× magnification with 2 × 2 camera binning. Individual cells were identified by Hoescht-positive nuclear staining. Lysotracker and Mitotracker were than quantitated in circles expanding 25 pixels outward from the nucleus.

### Histopathology

WT and *Cln6^nclf^* mice were killed, perfusion-fixed with PBS, and subsequently fixed with 4% PFA. Brains were sectioned on a vibratome at 50 μm (Leica VT10008). Sections were taken through standard DAB staining protocols [18]. Primary antibodies included anti-CD68 (AbD Serotec, MCA1957; 1:250), anti-GFAP (Dako, Z0334; 1:250), and anti-ATP synthase subunit C (Abcam, ab181243, 1:500). Secondary antibodies included anti-rat and anti-rabbit biotinylated antibodies (Vector Labs, 1:2000). Sections were imaged and analyzed using a Nikon 90i microscope with NIS-Elements Advanced Research software (V 4.20). Images were taken in the ventral posteromedial nucleus (VPM)/ventral posterolateral nucleus (VPL) of the thalamus and layers 5/6 of the somatosensory cortex, with three images taken per section and three sections imaged per mouse. For autofluorescent storage material, cells were considered positive for storage material when more than three autofluorescent puncta were aggregated around the nucleus. GFAP, CD68, and SubUnitC were analyzed using a threshold analysis in either NIS-Elements or ImageJ [18].

### Neurobehavior testing

#### Rotarod

WT and *Cln6^nclf^* mice were tested monthly (*n*=8–10; months 2–10) on a Rotamex-5 Rotarod (Columbus Instruments, Columbus, OH, U.S.A.) to assess motor capabilities. The machine was set to accelerate 0.3 revolutions per minute (rpm) every 2 s, with a starting speed of 0.3 rpm and a maximum speed of 36 rpm. Mice were trained for three consecutive trials, given a 30-min rest period, trained for three consecutive trials, given a second 30-min rest period, and trained for three final consecutive trials. After a 4-h rest period, mice were tested using the same paradigm as the training session. The latency to fall from the rotarod was averaged from each of the nine afternoon testing sessions to produce one value per mouse.

#### Water maze

Male WT and *Cln6^nclf^* mice were tested monthly (months 5–10) in a water maze apparatus to assess memory and learning deficiencies. The apparatus consisted of a 4-ft diameter tub filled with water to approximately 26 inches until the goal platform was submerged by 0.5 cm. The tub was aligned with four distinct visual cues on the walls of the tub at 0, 90, 180, and 270 degrees, with the platform resting in the maze at 315 degrees. Mice were trained first in a clear pool with a flagged platform. Mice were given 60 s to complete each trial, with four trials in the morning, followed by a 3-h rest period, and four additional trials in the afternoon. Mice that could not locate the platform with 50% accuracy in the time allotted were eliminated from further testing. Mice were then tested in water colored with white, non-toxic tempura paint and an un-flagged platform. Mice were given 60 s to complete each trial, with four trials in the morning, followed by a 3-h rest period, and four additional trials in the afternoon. Mice were tested for four consecutive days, each day starting at a different visual cue. Mice were recorded using Any-maze video tracking software (Stoelting Co., Wood Dale, IL, U.S.A.), and test duration, swim speed, and time spent along the walls were recorded and averaged from the 16 afternoon trials to produce one value per mouse.

### Statistics

For the *in vitro* experiments, an ordinary one-way ANOVA with an uncorrected Fisher’s LSD test was used comparing each group mean with the WT control group mean. For the immunohistochemistry and behavior experiments, an ordinary one-way ANOVA with a Bonferroni correction was used. Outliers were removed using the ROUT method Q = 0.1%. The survival analysis was performed using a log-rank Mantel–Cox test. Mean ± S.E.M. shown.

## Results

### (*S*)-Lacosamide improves lysosomal deficits in primary neuronal *Cln6* mutant cultures

**Figure 1:**
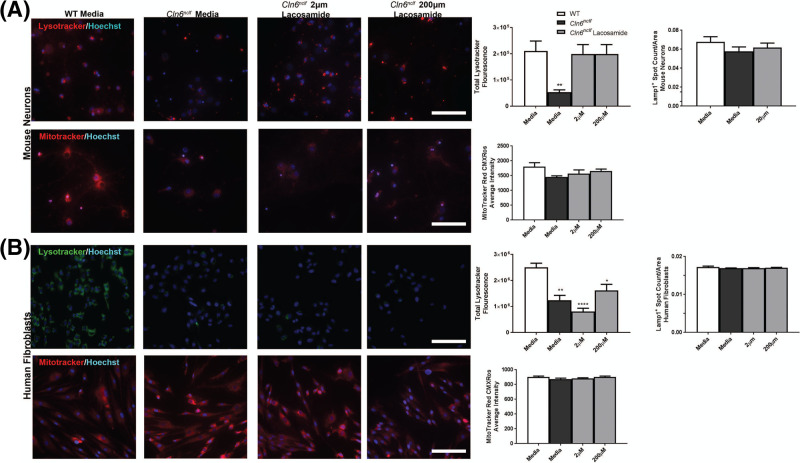
Treatment with (*S*)-Lacosamide rescues lysosomal deficits in *Cln6^nclf^* primary neuronal cultures (**A**) Treatment with 2 and 200 μM (*S*)-Lacosamide restores *Cln6^nclf^* mouse neuron lysosome pH abnormalities to WT levels, but has no effect on mitotracker abnormalities. *n*=5–6. Scale bar: 120 μm. LAMP1 lysosomal quantitation shown as a control for pH alterations (LCM: 20 μM). *n*=15–16. (**B**) Treatment with 200 μM (*S*)-Lacosamide partially restores *CLN6* patient fibroblast lysosome pH abnormalities to control levels, but has no effect on mitotracker abnormalities. *n*=3–9. Scale bar: 120 μm. LAMP1 lysosomal quantitation shown as a control for pH alterations (LCM: 2, 200 μM). *n*=6–15. For all analyses, one-way ANOVA with uncorrected Fisher’s LSD test, comparing *Cln6* mutant group means to WT control group mean was performed. Mean ± S.E.M.; *P<0.05, **P<0.01, ****P<0.0001.

To determine whether (*S*)-Lacosamide provides any cellular benefits in CLN6-Batten disease, patient fibroblast lines and neuronal mouse cultures were treated with varying doses of (*S*)-Lacosamide and examined 48 h later. As lysosomal and mitochondrial changes have been implicated in several forms of Batten disease, we focused on quantitating changes in these specific organelles. While *CLN6* patient fibroblasts and mouse neurons did not have alterations in LAMP1+ lysosomes, both cell types had reduced lysotracker fluorescence compared with WT neurons, indicating an increase in lysosomal pH. Treatment with 2 and 200 μM of (*S*)-Lacosamide rescued lysosomal pH deficits in *Cln6* mouse neurons, though it had a limited effect in *CLN6* patient fibroblasts (Figure 1A,B). In contrast, when both cell types were stained with MitoTracker Red CMXRos, a mitochondrial dye that is dependent on membrane potential to fluoresce, neither cell type had altered mitochondrial membrane potential compared with WT counterparts, as noted by their average fluorescent intensity (Figure 1A,B).

### (*S*)-Lacosamide alters glial activation in an acute, short-term treatment of *Cln6^nclf^* mice

**Figure 2:**
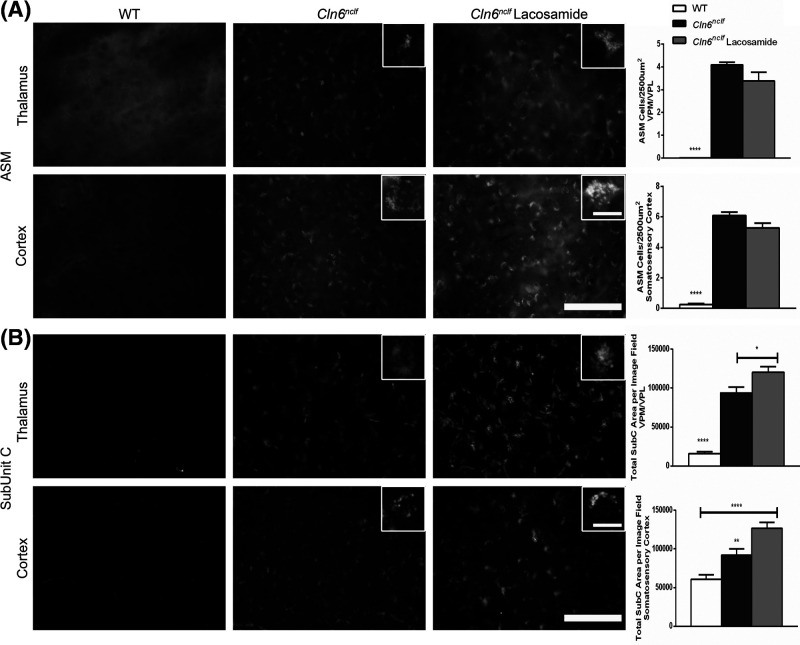
Acute treatment with (*S*)-Lacosamide does not reduce storage material burden in *Cln6^nclf^* mice (**A**) Acute treatment with (*S*)-Lacosamide does not reduce autofluorescent storage material (ASM) burden in the VPM/VPL or somatosensory cortex of *Cln6^nclf^*mice. (**B**) Acute treatment with (*S*)-Lacosamide increases SubunitC burden (SubC) in the VPM/VPL of *Cln6^nclf^* mice. ASM: *n*=7–8; Subunit C: *n*=28–42, biological *n*=7–8. One-way ANOVA, Bonferroni correction. Mean ± S.E.M.; *P<0.05, **P<0.01, ****P<0.0001. Scale bars: 50 μm. Inset scale bars: 8 μm.

**Figure 3:**
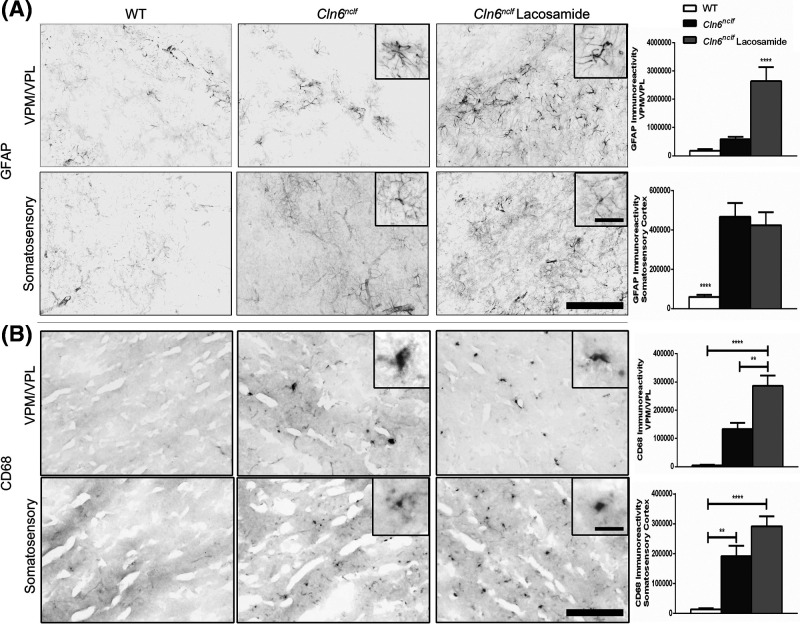
Acute treatment with (*S*)-Lacosamide increases glial activation in *Cln6^nclf^* mice (**A**) Acute treatment with (*S*)-Lacosamide increases astrocytic activation (GFAP) in the VPM/VPL of *Cln6^nclf^* mice, and has no effect in the somatosensory cortex. (**B**) Acute treatment with (*S*)-Lacosamide increases mitochondrial activation (CD68) in the VPM/VPL of *Cln6^nclf^* mice, and has no effect in the somatosensory cortex. GFAP: *n*=29–58, biological *n*=7–8; CD68: *n*=24–58, biological *n*=7–8. One-way ANOVA, Bonferroni correction. Mean ± S.E.M.; **P<0.01, ****P<0.0001. Scale bars: 50 μm. Inset scale bars: 8 μm.

As there were some benefits of treating *Cln6^nclf^* neurons with (*S*)-Lacosamide, 2-month-old WT and *Cln6^nclf^* mice were treated for 7 days with (*S*)-Lacosamide (20 mg/kg/day, i.p.) to determine the efficacy of the compound. This dose was based on previous studies indicating the compound was able to penetrate the brain through these and other methods, and that this dose effectively modulates CRMP2 phosphorylation [16,19,20]. Acute treatment with (*S*)-Lacosamide did not affect the number of cells with accumulated autofluorescent storage material at 3 months of age, and appeared to increase the amount of SubunitC accumulation (Figure 2A,B). Similarly, acute treatment with (*S*)-Lacosamide increased both astrocyte and microglial activation in the VPM/VPL, and had no effect on the somatosensory cortex of *Cln6^nclf^* mice (Figure 3A,B). While these data were not entirely encouraging, as we would anticipate an efficacious compound reducing these pathological markers, we recognize the possibility of enhanced glial activation being beneficial to neurodegeneration [21]. We therefore determined the effect of sustained treatment of (*S*)-Lacosamide on behavioral and pathological deficits in *Cln6^nclf^* mice.

### Acute treatment of (*S*)-Lacosamide has no positive effects on the long-term behavior or pathology of *Cln6^nclf^* mice

**Figure 4:**
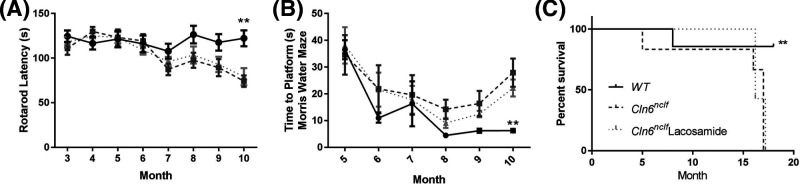
Sustained treatment with (*S*)-Lacosamide does not improve motor, memory/learning, or survival deficits in *Cln6^nclf^* mice (**A**) Sustained treatment with (*S*)-Lacosamide does not improve motor deficits in *Cln6^nclf^* mice in an accelerating rotarod test at 10 months of age. (**B**) Sustained treatment with (*S*)-Lacosamide does not improve memory and learning deficits in *Cln6^nclf^* mice in a Morris water maze test at 10 months of age. (**C**) Sustained treatment with (*S*)-Lacosamide does not improve survival deficits in *Cln6^nclf^* mice. *n*=8–10. One-way ANOVA, Bonferroni correction for rotarod, and Morris water maze. Log-rank (Mantel–Cox) test for survival curve. Mean ± S.E.M.; **P<0.01.

**Figure 5:**
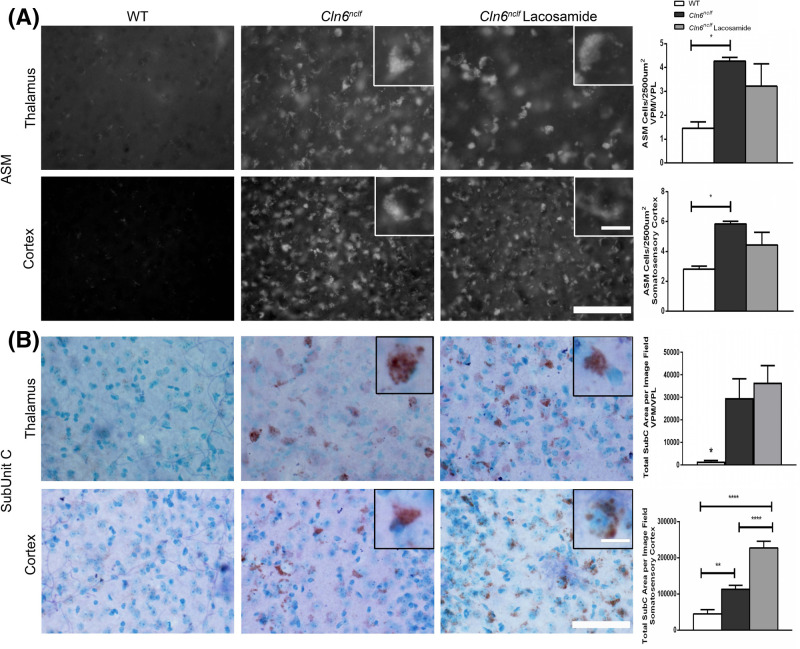
Sustained treatment with (*S*)-Lacosamide does not reduce storage material burden long term in *Cln6^nclf^* mice (**A**) Sustained treatment with (*S*)-Lacosamide does not reduce autofluorescent storage material (ASM) burden in the VPM/VPL or somatosensory cortex of *Cln6^nclf^* mice. (**B**) Sustained treatment with (*S*)-Lacosamide increases SubunitC burden (SubC) in the somatosensory cortex of *Cln6^nclf^* mice. Blue counterstain represents Methyl Green, brown DAB stain represents subunit C reactivity. ASM: *n*=3; Subunit C: *n*=12–33, biological *n*=3. One-way ANOVA, Bonferroni correction. Mean ± S.E.M.; *P<0.05, **P<0.01, ****P<0.0001. Scale bars: 50 μm. Inset scale bars: 8 μm.

**Figure 6:**
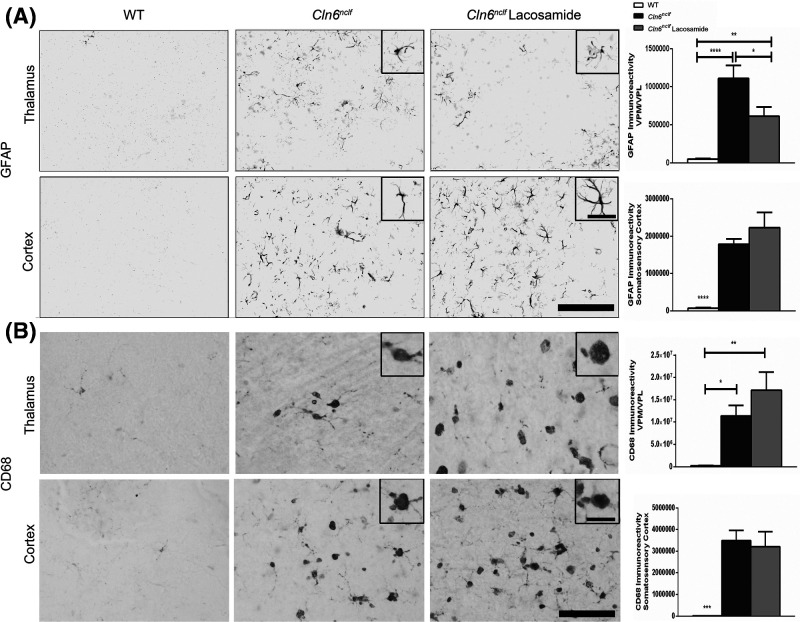
Sustained treatment with (*S*)-Lacosamide reduces astrocyte activation long term in the VPM/VPL of *Cln6^nclf^* mice, but does not affect any other glial activation (**A**) Sustained treatment with (*S*)-Lacosamide reduces astrocytic activation (GFAP) in the VPM/VPL of *Cln6^nclf^* mice, and has no effect on GFAP reactivity in the somatosensory cortex. (**B**) Sustained treatment with (*S*)-Lacosamide does not affect microglial activation (CD68) in the VPM/VPL or somatosensory cortex of *Cln6^nclf^* mice. GFAP: *n*=24–27, biological *n*=3; CD68: *n*=11–27, biological *n*=3. One-way ANOVA, Bonferroni correction. Mean ± S.E.M.; *P<0.05, **P<0.01, ***P<0.001, ****P<0.0001. Scale bars: 50 μm. Inset scale bars: 8 μm.

Two-month-old WT and *Cln6^nclf^* mice were treated daily with (*S*)-Lacosamide for 3 months at 20 mg/kg/day (i.p.) and monitored for behavioral and survival outcomes. Sustained treatment with (*S*)-Lacosamide did not improve motor, memory/learning, or survival deficits in *Cln6^nclf^* mice (Figure 4A–C). Similarly, there were no improvements in accumulation of autofluorescent storage material in the brains of treated 11-month-old animals, although there was a sustained increase in SubunitC in the somatosensory cortex (Figure 5A,B). Alternatively, sustained treatment with (*S*)-Lacosamide produced a moderate reduction in astrocyte reactivity (GFAP) in the VPM/VPL of 11-month-old brains, contrary to what was seen with the acute treatment, and had no effect on astrocyte reactivity in the somatosensory cortex, or microglial reactivity in either area of the brain (Figure 6A,B). Nevertheless, these alterations in subunit C and GFAP reactivity did not have a functional benefit or detriment in *Cln6^nclf^* mice.

## Discussion

Here, we broadly explored the therapeutic potential of CRMP2 modulation in a mouse model of CLN6-Batten disease. While CRMP2 has been studied rather extensively in its role in neuronal trafficking, polarization, and differentiation, and has been associated with CLN6 and Batten disease features, there has been limited study in its use as a therapeutic target in neurodegenerative diseases [7,8]. CRMP2-targeted therapies have been explored in multiple sclerosis mouse models to some success, and have been used to relieve chronic neuropathic pain by blocking CRMP2’s binding to calcium voltage-gated channels [22–24] or by allosteric regulation of voltage-gated NaV1.7 channels by CRMP2 [25–27]. There are several studies suggesting dysregulated CRMP2 dynamics in Alzheimer’s disease and schizophrenia, yet it is unclear whether this is causal of disease presentation or simply a secondary effect of disease outcome [9–11].

While (*S*)-Lacosamide has been previously shown to reduce p-CRMP2 levels at the studied dose [16], the phosphorylation status of CRMP2 in Batten disease has not been explored, though the total level of CRMP2 is reduced in *Cln6^nclf^* brains [7,16]. Further, in addition to reducing p-CRMP2 levels, (*S*)-Lacosamide has been shown to reduce CRMP2’s ability to promote neurite outgrowth in primary rat cortical neurons [28]. While our study did not show any detrimental functional effects of (*S*)-Lacosamide administration in *Cln6^nclf^* mice, it is possible that this mode of action of (*S*)-Lacosamide may have played a role in the compound being non-therapeutic.

Interestingly, while the (*R*) enantiomer has been reported to decrease glial activation in a number of contexts, acute treatment with the (*S*) enantiomer increased astrocyte activation in 3-month-old *Cln6^nclf^* animals [14,29]. Enantiomers have long been known to produce different pharmacological results, due to varying absorption, distribution, and metabolism rates [30–32]. However, (*S*)-Lacosamide has only been shown to be less efficacious than the *R* enantiomer in reducing seizure activity. While the (*S*) enantiomer has been shown to effectively target CRMP2, its efficacy in other effects has not been well studied [33]. As such, an early increase in glial activation could potentially be neuroprotective if it leads to restoration of damaged processes, such as rescue of lysosomal or synaptic dysfunction in Batten disease [21]. However, it is also possible that a sustained increase in glial activation could further exacerbate any neurodegenerative processes [21]. Conversely, with sustained (*S*)-Lacosamide treatment, astrocyte activation was moderately reduced in 11-month-old *Cln6^nclf^* mice. While this could be a marker of slowed disease progression, this ultimately did not translate to any functional benefit in the *Cln6^nclf^* animals. As such, further studies are needed to determine the full extent of the therapeutic differences of (*R*,*S*)-Lacosamide, as well as the effect of glial activation on the progression on Batten disease.

In particular, our study is merely descriptive of the therapeutic utility of (*S*)-Lacosamide in CLN6-Batten disease. The inefficacy of (*S*)-Lacosamide could be due to the low daily dose (20 mg/kg) used in our experiments. (*S*)-Lacosamide, given intraperitoneally, has an ED_50_ of 100–300 mg/kg in the maximal electroshock seizure test and a TD_50_ (i.e. toxic dose, determined from the rotarod test) of >300 mg/kg [34]. Since the half-life of (*S*)-Lacosamide is ∼3 h and peak levels are achieved within ∼40 min of an oral administration [20], it is likely that once daily dosing is insufficient to reach a stable level of the drug to achieve stable silencing of CRMP2 activity, a fact compounded by the ∼6 h half-life of the CRMP2 protein itself. While (*S*)-Lacosamide’s mechanism of action in regard to CRMP2 modulation has been extensively studied by our group, further work on the mechanism by which the compound failed to be efficacious in this particular disease model remains to be done. Future studies will utilize an oral administration route to maximize stable drug delivery, with (*S*)-Lacosamide incorporated in AIN93M chow (Research Diets, New Brunswick, NJ) at >100 ppm, based on pilot studies showing no adverse effects at 100 ppm (data not shown). We anticipate an average daily intake will be 6 g chow per 25 g mouse, giving an average daily dose of 24 mg (S)-LCM/kg. Even with this dose and route optimization, however, the use of CRMP2-targeted therapies in neurological disorders is ultimately a newly developing field [6], and is even less developed in the context of Batten disease.

In conclusion, we sought to test whether modulation of CRMP2 activity would prove to be therapeutic in a mouse model of CLN6-Batten disease. Our results suggest that while administration of (*S*)-Lacosamide rescued lysosomal abnormalities *in vitro* and altered glial activity *in vivo*, the compound ultimately did not have any behavioral or survival benefits in *Cln6^nclf^* mice. As such, alteration of CRMP2 activity via (*S*)-Lacosamide alone is unlikely to be a viable therapeutic target for CLN6-Batten disease, though it may prove to be beneficial when combined with other, synergistic compounds.

## Abbreviations

ANOVA: analysis of variance
CLN6: ceroid-lipofuscinosis neuronal protein 6
CRMP2: collapsing response mediator protein 2
DAB: diaminobenzidine
ER: endoplasmic reticulum
GFAP: glial fibrillary acidic protein
HBSS: Hank’s Balanced Salt Solution
i.p.: intraperitoneal
LAMP1: lysosomal associated membrane protein 1
LSD: least significant difference
PFA: paraformaldehyde
rpm: revolutions per minute
SubunitC: mitochondrial ATP synthase subunit C
(*S*)-Lacosamide: *S*-N-benzy-2-acetamido-3-methoxypropionamide
VPM/VPL: ventral posteromedial nucleus/ventral posterolateral nucleus
WT: wild-type
